# Human Health Risk Assessment and Potentially Harmful Element Contents in the Cereals Cultivated on Agricultural Soils

**DOI:** 10.3390/ijerph17051674

**Published:** 2020-03-04

**Authors:** Agnieszka Gruszecka-Kosowska

**Affiliations:** Department of Environmental Protection, Faculty of Geology, Geophysics, and Environmental Protection, AGH University of Science and Technology, Al. Mickiewicza 30, 30-059 Kraków, Poland; agnieszka.gruszecka@agh.edu.pl

**Keywords:** potentially harmful elements, cereals, agricultural soil, human health risk assessment, bioconcentration factor, dietary intake

## Abstract

Potentially harmful element (PHE) contents were investigated in six species of cereals in southern Poland, with human health risk implications assessed afterwards. The PHE contents belonged to the following ranges (mg/kg wet weight): As below the limit of detection (<LOD)–0.013, Cd <LOD–0.291, Co <LOD–0.012, Cu 0.002–11.0, Hg <LOD–0.080, Ni <LOD–8.40, Pb <LOD–12.0, Sb <LOD–0.430, Tl <LOD–0.160, and Zn 5.47–67.7. The Pb and Cd contents exceeded the maximum allowable concentration (MAC) values for wheat, oat, rye, and barley in the Śląskie region. The bioaccumulation coefficient (BA) for the total PHE content in the soil indicated that cereals had no potential of PHE accumulation. Regarding the statistical daily consumption of cereals, the PHE intake rates, expressed as a percentage of permissible maximum total daily intake (% PMTDI), were the following: As 0.0003, Cd 0.193, Co 0.0003, Cu 0.075, Hg 0.424, Ni 3.94, Pb 3.16, Sb 0.23, Tl 0.27, and Zn 0.44. The total non-carcinogenic risk values (HQ) exceeded the target risk value of 1 for wheat (HQ = 13.3) and rye (HQ = 3.44). For other cereals, the total non-carcinogenic risk values decreased in the following order: barley (HQ = 0.47) > oat (HQ = 0.38) > maize (HQ = 0.02). The total non-carcinogenic risk value of the statistical daily consumption of cereals was acceptable low (HQ = 0.58). The acceptable cancer risk (CR) level of 1.0 × 10^−5^ investigated only for As was not exceeded under any of the intake scenarios. Concerning the mean As content in cereals consumed daily in statistical amounts the CR value was equal to 5.1 × 10^−8^. The health risk value according to the Pb content in cereals using the margin of exposure (MOE) approach was equal to 1.27, indicating an acceptable low risk.

## 1. Introduction

Cereals are the staple food in the human diet of the population worldwide. Globally, the daily consumption of cereals (403 g/person per day) is higher than that of vegetables (385 g/person per day) and fruits (213 g/person per day) [1]. Cereals and breads are the main source of energy in all age groups, and they contribute 31% of digestible energy to adults [2]. Each cereal plays an important role in various food industry areas: barley is used in beer production, wheat and rye are used in bakery products, oats are used in oatmeal and muesli, and triticale, besides being the main ingredient of animal feed, is also used on a smaller scale in bread, cookies, or cakes [3]. In the nutritional pyramid, the healthy diet comprises from four to six servings of grains daily [4]. Cereals are substantial sources of carbohydrates, dietary fiber, water-soluble vitamins, minerals, proteins, amino acids, phytochemicals (phenolics and terpenoids), and methyl donors (betaine, choline) [1,2]. 

For many years, so-called heavy metals have been contaminants of special concern due to their persistence in the environment and their ability to accumulate in ground or soil. Due to the fact that metalloids are included among them, the term “trace elements” is also used [5]. However, some trace elements are also essential for living cells, i.e., micronutrients such as Zn, Cu, Co, Cr (III), and Ni, depending on the organism [6]. Nevertheless, as the dose makes the poison, quoting Paracelsus, intake of higher amounts of essential elements than needed can cause the occurrence of negative health effects [7,8]. On the other hand, some trace elements are redundant for living organisms; thus, any dose may cause toxic effects. Hence, the term potentially harmful elements (PHEs) takes into account all of the above [9]. Exposure to PHEs and their accumulation in humans may cause many toxic health effects, including reproductive toxicity, neurotoxicity, immune suppression, birth defects, and behavioral or developmental changes [10,11]. PHEs are also involved in the emergence of such diseases as Alzheimer’s disease, Parkinson’s disease, osteoporosis, or multiple sclerosis [12]. Moreover, some of them are considered to be carcinogenic to humans, i.e., As, Cd, Cr, Co, Ni, and Pb [13].

As the environment becomes more and more polluted with PHEs in agriculture, especially with fertilizers, fungicides, and agrochemicals used for crop protection purposes, as well as rapidly expanding urban and industrial areas and related atmospheric contaminant depositions [14,15,16,17], soil and food crop quality has become a serious health issue worldwide [12,18,19,20,21,22,23,24,25,26,27]. Moreover, special concern has started to be paid to baby foods, as recent research of Houlihan and Brody [28] revealed that 95% of baby foods were contaminated by at least one of the heavy metals, and the most common heavy metals in baby foods were As, Cd, Hg, and Pb.

Cereals are the main components of the daily diet. Thus, it is justified to hypothesize that the intake of seeds containing even low concentrations of PHEs may cause health risks owing to long-term consumption of cereals. Research of arable soils in southern Poland, where investigated cereals were cultivated, revealed that total permissible PHE contents were not exceeded in these soils [29]. However, bioavailable PHE contents were found in investigated soils [30]. The precise use of fertilizers, particularly in the analyzed soils, was not known, however due to rather weak soil production properties in Poland [31], mineral and organic fertilizers, and the food protection products are commonly used in Polish agriculture [32]. Moreover, vegetables, investigated precisely on these analyzed soils, were enriched in PHEs, and non-carcinogenic health risks related to their consumption exceeded the target risk value of 1 [33]. In the case of cultivated fruits on investigated soils, it was revealed that berry, stone, and pome fruits had the potential to accumulate PHEs [34]. Thus, in the case of cereals, which are the major source of daily diet composition, the current research seemed to be important and justified. 

The goal of the present study was to determine selected PHE concentrations in commonly cultivated cereals on arable soils in southern Poland and to conduct a health risk implication assessment. The detailed objectives of the study included the following: (1) the determination of As, Cd, Co, Cr, Cu, Hg, Ni, Pb, Sb, Se, Tl, and Zn contents in six species of cereals (barley, maize, oat, rye, triticale, and wheat), (2) the calculation of soil-to-plant transfer indices, (3) the determination of PHE contributions to the daily intake rates via cereal consumption, and (4) a human health risk assessment of the PHE contents in consumed cereals.

## 2. Materials and Methods 

### 2.1. Cereal Sampling and Preparation

**Figure 1:**
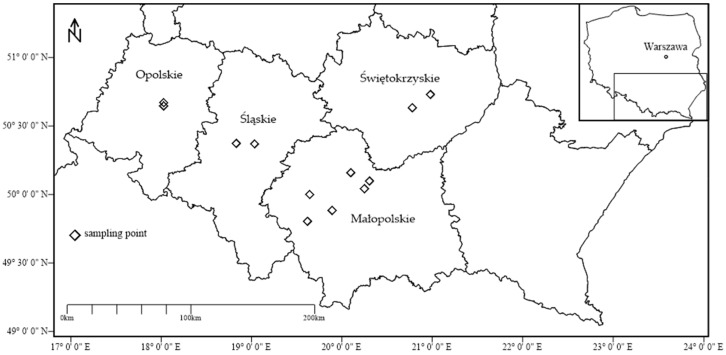
Cereal sampling site locations in the four regions of southern Poland (modified from [34]).

Previously, total [29] and bioavailable [30] contents of potentially harmful elements were investigated in arable soils of southern Poland. Subsequently, edible plants commonly cultivated in Poland on the above-described soils were further analyzed and described with respect to vegetables [33] and fruits [34]. The results presented in this paper focus on cereal investigations. In the four regions of southern Poland, namely Opolskie, Śląskie, Małopolskie, and Świętokrzyskie (Figure 1), during the growing seasons of 2015 and 2016, six species of cereals commonly cultivated in Poland were investigated (Table 1). Cereal samples were obtained directly from farmers. Not all the cereal species were collected in all investigated regions due to the research assumption that cereals had to be cultivated directly on arable soils previously investigated [29,30]. Thus, altogether 18 cereal samples were analyzed. After transportation to the laboratory, cereals were washed, and seeds were placed on open porcelain dishes and dried under radiant lamps at 70 °C. Dried cereal samples were then ground into coarse powder using a ceramic coffee grinder and stored in sealed bags for further analysis. The grinder was cleaned after each cereal sample processing to prevent cross-contamination. 

### 2.2. Sample Analyses

Cereal samples (0.5 g, accurate to 0.001 g) were weighed and placed in mineralization flasks, with 15 mL of HNO_3_ and 5 mL of H_2_O_2_ added. Samples were left for decomposition of organic matter overnight. The next step involved digestion in the SCP Science DigiPREP HT High Temperature Digestion System (SCP Science, Quebec, Canada) for 2 h at 130 °C. After cooling down the extract solutions, their volume was expanded to 50 mL using ultrapure water. At the same time, blank and reference samples (white cabbage, Certified Reference Material BCR^®^-679, European Commission, Joint Research Centre, Institute for Reference Materials and Measurements) were prepared. The total concentrations of the investigated PHEs (As, Cd, Co, Cr, Cu, Hg, Ni, Pb, Sb, Se, Tl, and Zn) were determined by inductively coupled plasma-mass spectrometry ICP-MS (ELAN 6100; Perkin Elmer, Waltham, MA, USA), according to United States Environmental Protection Agency (USEPA) 6020B [35] and ISO 17294-2:2003 [36] protocols. 

All the results of the PHE concentrations in the cereals were referred to by wet weight (ww), in accordance with Equation (1) [37]. Water content in particular plant samples was taken from the USEPA exposure factors book [37]:

c_ww_ = c_dw_ × (100 − w)/100 (1)

where c_ww_—concentration of PHE in plant sample wet weight, c_dw_—concentration of PHE in plant sample dry weight, and w—percentage water content. 

### 2.3. Quality Control

Cereal sample analyses were performed, observing standard certified analytical quality control procedure ISO 17294-1:2007 [38]. To achieve impartial and unequivocal ICP-MS results, all investigated PHEs were also measured using inductively coupled plasma-optical emission spectroscopy ICP-OES (OPTIMA 7300DV; Perkin Elmer, Waltham, MA, USA) according to USEPA 6020B [35] and ISO 11885:2009 [39] protocols. The certified reference material (white cabbage, Certified Reference Material BCR^®^-679) was analyzed at the same time. Recovery from the Certified Reference Material BCR^®^-679 was between 83% and 124% for the majority of the analyzed PHEs. Method reagent blanks and duplicates were used to provide quality assurance and quality control. All the reagents used in the laboratory analysis were analytically pure. The results of the samples were contained within the allowable error change values. Analytical bias was statistically insignificant (*p* = 0.05). Precision measurements of the ICP-MS and ICP-OES systems were satisfactory, verified by six different solution injections. Rh was used as an internal standard. Element correction equations were used for each element so that the ICP-MS analysis minimized the impact of interferences. The limit of detection of the instrument (LOD) values of the investigated PHEs were as follows (µg/dm^3^): As < 0.001, Cd < 0.0005, Co < 0.0005, Cr < 0.0005, Cu < 0.0005, Hg < 0.001, Ni < 0.002, Pb < 0.0005, Sb < 0.0005, Se < 0.002, Tl < 0.001, and Zn < 0.001.

### 2.4. Statistical Analysis

Statistical analysis involved the determination of mean, standard deviation, and minimum and maximum values using a Microsoft Excel 2007 spreadsheet. The software package STATISTICA 13 (TIBCO Software Inc., Palo Alto, CA, USA) was used to check data distribution using the Shapiro–Wilk’s test (*p* = 0.05), while hierarchical cluster analysis (HCA) and principal component analysis (PCA) were performed for the multivariate statistical modeling of the input data. Halves of the limit of detection values were assigned to undetected results in all performed statistical analyses, as recommended by the World Health Organization (WHO) [40]. 

### 2.5. Soil-To-Plant Transfer Indices

Two soil-to-plant transfer factors were used in the current research for investigating if determined total and bioavailable PHE contents in analyzed soils significantly affected their concentrations in consumed cereal seeds. The bioaccumulation coefficient (BA) described the transference of PHE from soil to plant, while the bioconcentration coefficient (BC) described plant capacity to adsorb PHE from soil when PHE occurred in an available form [41]. The BA and BC values were calculated for the investigated groups of cereals, respectively, from Equations (2) and (3) according to [42]:

BA = C_cereals_/C_st_ (2)

BC = C_cereals_/C_sa_ (3)

where C_cereals_ is the mean concentration of a particular PHE (mg/kg ww) in the investigated cereal seeds; C_st_ is the mean total concentration of a particular PHE, determined in the soil samples (mg/kg dw) from southern Poland, using aqua regia digestion [29]; and C_sa_ is the mean available concentration of a particular PHE, determined in the soil samples (mg/kg dw) from southern Poland after extraction with the following: (i) 0.11 M CH_3_COOH (the first fraction of the Community Bureau of Reference BCR sequential extraction procedure F1) and (ii) 0.05 mol/dm^3^ Na_2_EDTA [30]. The contents of PHEs used for calculations of soil-to-plant transfer indices are given combined in Table S1 based on previous soil investigations [29,30].

### 2.6. Human Health Risk Assessment

The point estimate method developed by the USEPA [43] was applied to assess the human health risk analysis (HHRA) arising from the consumption of PHEs in the cereals cultivated in southern Poland. Using the mean and the 95th percentile (P95) of the PHE concentrations in the cereals, the following parameters were calculated for both adults and children: the daily intake rate (DIR) values for particular PHEs were calculated as the sum of consumed cereals according to Equation (4) [44]:

DIR = Σ (C_cereals_ × IR_cereals_/BW) (4)

where C_cereals_ is the concentration of a particular PHE in the group of cereals (mg/kg ww); IR is the cereals ingestion rate (g/person per day) in the group of cereals; and BW is body weight: 70 kg for adults and 15 kg for children [37]. 

The intake rates of cereals assumed in this study are presented in Table 2. Three exposure scenarios of cereal consumption were analyzed. The first scenario was based on the available Polish data concerning statistical cereal consumption by adults [45]. The second and third ones were based on the recommended number of servings of cereals per day for adults and children, respectively [4]. The recommended values [4] describe the amounts of cereals that should be consumed daily when one follows healthy diet suggestions. As, in fact, these amounts of cereals are still rarely consumed, they were also chosen to represent the highest amount of cereals consumed in the calculated HHRA analysis performed.

The average daily doses (ADD) for PHE ingestion via consumed cereals (mg/kg bw per day) were calculated as the sum of the consumed cereals using Equation (5) according to [43]:

ADD = Σ (C_cereals_ × IR_cereals_ × EF × ED × 10^−3^) / AT × BW (5)

where C_cereals_ is the PHE concentration in the investigated cereals (mg/kg ww); IR_cereals_ is the intake rate of cereals (g/person per day); EF is the exposure frequency: 365 d/y; ED is the exposure duration: 30 y for adults and six years for children [37]; AT is the averaging time in days: ED × 365 for non-carcinogens and 70 y × 365 for carcinogens [46]; BW is body weight (kg), as in Equation (4); and 10^−3^ is the unit conversion factor. 

The non-carcinogenic PHE risk values from dietary exposure were calculated with Equation (6), according to [43]:

HQ = ADD/RfD(6)

where HQ is the hazard quotient and RfD is the reference dose for a particular PHE. 

The RfD values were set as follows (mg/kg bw per day): As 3.0 × 10^−4^, Cd 1.0 × 10^−3^, Co 3.0 × 10^−4^, Cu 4.0 × 10^−2^, Hg 3.0 × 10^−4^, Ni 2.0 × 10^−2^, Sb 4.0 × 10^−4^, Tl 1.0 × 10^−5^, and Zn 3.0 × 10^−1^ [47]. 

The total non-carcinogenic risk (HQt) value for the investigated PHEs was calculated using Equation (7) according to [43]:

HQt = HQ_1_ + HQ_2_ + … + HQ_n_ (7)

where HQ are the hazard quotient values for 1-n PHEs investigated in the study. 

The carcinogenic risk values of PHEs from dietary exposure were calculated using Equation (8) according to [43]:

CR = ADD × SF_o_ (8)

where CR is the carcinogenic risk and SF_o_ is the oral slope factor for a particular PHE. 

Only As was considered to be a carcinogenic PHE in this study. There were no SF values available for other investigated elements at the time. The SF_o_ for As was set to be equal to 1.5 (mg/kg bw per day)^−1^ [47]. The total carcinogenic risk value, as the sum of partial CR values, was not calculated because As was the only carcinogenic PHE considered in this study. 

The Pb risk of dietary exposure was calculated according to the margin of exposure (MOE) approach, as recommended by the European Food Safety Authority (EFSA) [48], using Equation (9) [49]:

MOE = BMDL/DIR(9)

where MOE is the margin of exposure value; BMDL is the benchmark dose (or the lower confidence limit), estimated at 1.2 µg/kg bw per day for adults and 0.6 µg/kg bw per day [17]; and DIR is the total amount of cereals consumed daily under the analyzed intake scenarios. 

## 3. Results

### 3.1. PHE Contents in Cereals

In the six investigated species of cereals, the concentrations of Cr and Se were <LOD, and thus, those two potentially harmful elements were excluded from further analysis. The detectable rates in cereal samples (%) of remaining PHEs were the following: As 6.25%, Cd 69.2%, Co 6.25%, Cu 100%, Hg 46.8%, Ni 76.9%, Pb 30.8%, Sb 76.9%, Tl 38.5%, and Zn 100%. The concentrations of the remaining 10 PHEs in the investigated cereals (Table 3) were contained within the following ranges (mg/kg ww): As <LOD–0.013, Cd <LOD–0.291, Co <LOD–0.012, Cu 0.002–11.0, Hg <LOD–0.080, Ni <LOD–8.40, Pb <LOD–12.0, Sb <LOD–0.430, Tl <LOD–0.160, and Zn 5.47–67.7.

The results of the PHE contents in barley revealed the following decreasing order: Zn > Ni > Pb > Cu > Cd > Sb > Tl. For maize, the decreasing order of PHE contents was as follows: Zn > Ni > Cu > Sb > Cd > As > Co > Hg > Pb > Tl. In oat, the decreasing order of PHE contents was the following: Zn > Pb > Cu > Ni > Tl > Sb. The decreasing order of PHE contents in rye was as follows: Zn > Cu > Pb > Ni > Sb > Tl. For triticale, the decreasing order of PHE contents was the following: Zn > Cu > Ni > Sb > Tl > Cd. For wheat, the decreasing order of PHE contents was as follows: Zn > Cu > Ni > Pb > Sb > Hg > Cd > Tl. If any PHEs were not mentioned in the abovementioned sequences, their contents were <LOD in a particular cereal. Considering essential elements, the decreasing order of Cu in the investigated cereal species was as follows: rye > oat > wheat > barley > triticale > maize. In the case of Zn, its decreasing order in the cereals was the following: rye > oat > barley > wheat > triticale > maize. For nonessential elements, the decreasing order of particular PHEs in the investigated cereal species was as follows: As: maize > barley > oat > rye > triticale > wheat; Cd: barley > wheat > maize > triticale > oat > rye; Co: maize > barley > oat > rye > triticale > wheat; Hg: wheat > maize; Ni: barley > wheat > triticale > maize rye > oat; Pb: oat > rye > barley > wheat > maize > triticale; Sb: rye > wheat > barley > oat > maize > triticale; and Tl: oat > rye > triticale > barley > wheat > maize. 

In order to determine the analyzed cereal safety, the PHE content values were compared to the permissible levels stated in the EU regulation on maximum levels for certain contaminants in foodstuff [50]. The maximum allowable concentration values in cereals were specified for two investigated PHEs (mg/kg ww): Pb 0.20 and Cd 0.10 (for wheat: 0.20). The contents of Pb and Cd significantly exceeded the MAC values in wheat, oat, rye, and barley in the Śląskie region. In the analyzed regions of southern Poland, the Pb and Cd contents in maize were much lower than the MAC values. The Pb and Cd contents in all the analyzed samples of triticale were <LOD. 

### 3.2. Statistical Analysis

PCA performed on the PHE concentrations in the investigated 18 cereal samples collected in southern Poland revealed three principal components (PCs), with eigenvalues higher than 1, which altogether explained 84.2% of the variance observed (Table 4). The first principal component (PC1) that accounted for 44.4% of variance had high (>0.6) positive loading values of As and Co that were probably caused by the fact that As and Co contents were determined only in maize samples. On the other hand, the high negative loading values of Cu, Pb, Sb, and Zn indicated that the lowest contents of those PHEs were stated in maize, as compared to the remaining investigated cereal species. The second principal component (PC2) explained 26.0% of variance and had negative loading values for Ni and Cd, indicating a tendency of rye and oat to contain those PHEs. Moreover, the component could be correlated with easiness of Cd and Ni bioaccumulation in plants [51,52,53,54], because bioavailable concentrations of those PHEs were determined in soil [30]. The PCA ordination diagram for the first two PCs computed for PHE contents in cereals is presented in Figure S1. The third principal component (PC3) explained 13.8% of variance and had a high negative loading value for Tl. That might be associated with other than geogenic sources of Tl, such as from the atmospheric deposition as described in [55,56], while a positive factor of the loading value for Sb might indicate mainly geogenic sources of Sb, as the function of concentration in parent rocks and non-ferrous ores occurring in the soils where the cereals were cultivated [5,57]. In the cases of the remaining PHEs, their uptake by plants might come from both sources: as the effect of natural occurrence in soil and as the anthropogenic activity related to mining and processing Zn-Pb and Cu ores. 

HCA explored similarities between the contents of the investigated PHEs in the analyzed cereals. A hierarchical dendogram distinguished three similarity groups for the variables according to Sneath’s criterion equal to 1/3 D_max_ (Figure S2). Cluster I could be associated with maize, as this cereal contained the highest contents of non-essential PHEs, i.e., As and Co, and simultaneously the lowest contents of essential PHEs, i.e., Cu and Zn. Cluster II could be associated with rye, as that cereal contained the highest proportions of Zn and Cu among the investigated cereals. Cluster III was connected with oat, triticale, wheat, and barley, because the investigated contents of PHEs were placed between the values observed in Clusters I and II. Standardized contents of PHEs in the regions of southern Poland were also presented on a color scale map (Figure S3) to show the geological background and soil impact on the PHE uptake. The map revealed that the highest quantities of Pb, Cd, Ni, Cu, Tl, and Zn, in comparison to other regions, were observed in the investigated cereals (except for maize) sampled in the Śląskie region. The highest quantities of As and Co were observed in maize sampled in the Małopolskie region.

### 3.3. Soil-To-Plant Transfer Indices

**Figure 2:**
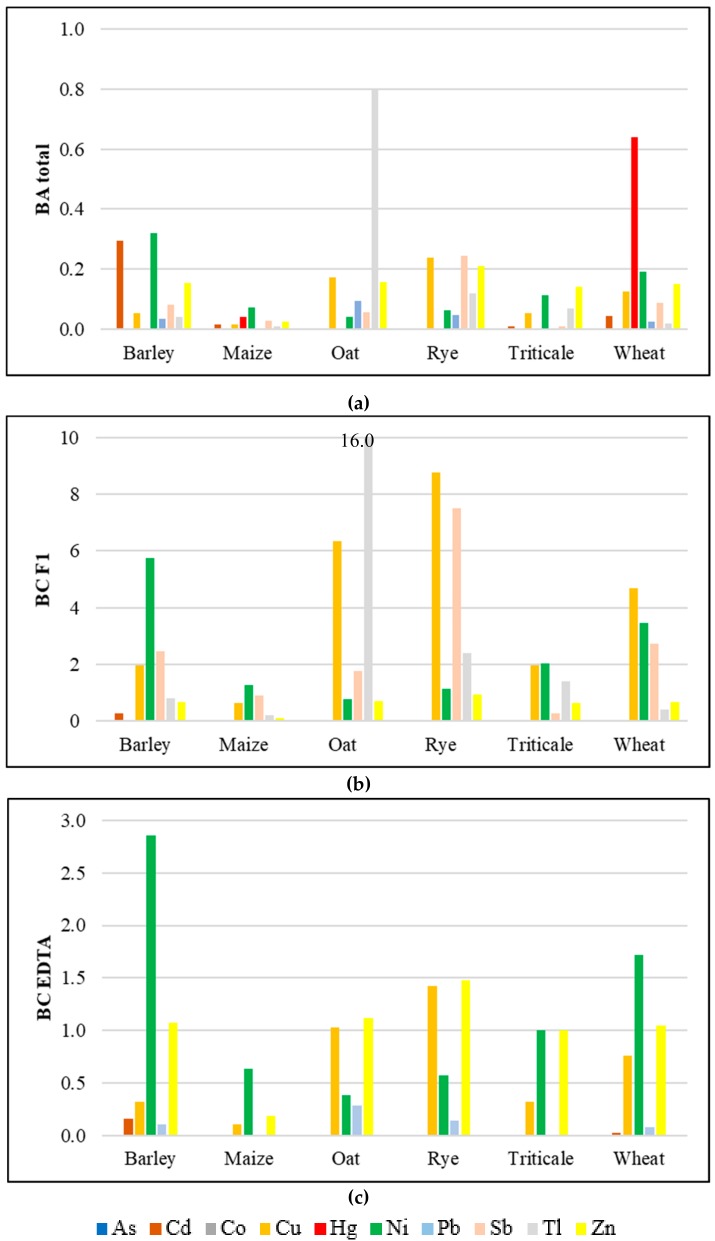
Soil-to-plant transfer indices for the investigated groups of cereals: (**a**) BA_total_—bioaccumulation coefficient, total PHE content; (**b**) BC_F1_—bioconcentration coefficient, PHE content of the F1 fraction of the BCR sequential extraction; (**c**) BC_EDTA_—bioconcentration coefficient, PHE content 0.05 mol/dm^3^ of the Na_2_EDTA extraction.

Because plants stock up elements mainly from soil, transfer indices are used to determine the efficiency of the PHE uptake. In this study, bioaccumulation and bioconcentration indices were calculated for the investigated cereals cultivated in the regions of southern Poland. The numerical data of the distribution of the transfer-index calculated values were presented on the box-and-whisker chart in Figure 2. The bioconcentration coefficient (BC_F1_) values were not calculated for Hg and Pb, because those PHE contents in soil samples had been <LOD after the first step of the BCR extraction procedure. The bioconcentration coefficient (BC_EDTA_) values were also not calculated for As, Co, Hg, Sb, or Tl, because those PHEs had not been determined in soil samples after the 0.05 mol/dm^3^ Na_2_EDTA extraction procedure. Moreover, the bioaccumulation coefficient (BA_total_) values were not calculated for Cr or Se, because 100% of the cereal samples indicated PHE concentrations of <LOD. The calculated BA_total_ values were <1 (Figure 2a) in the cases of all cereals, which indicated that the investigated grains had no potential of PHE accumulation; however, the highest accumulation capacity was observed for Tl in oat (BA = 0.8) and for Hg in wheat (BA = 0.64). This could be associated with the concentration of those PHEs in soil, as well as with the ease of uptake from soil and transportation in plant tissues; however, grains are the most protected parts against harmful substances in the plant [5]. The calculated BC_EDTA_ values indicated the potential for PHE accumulation in cereals (BC values >1) (Figure 2c) for Ni > Zn in barley, Zn > Cu in oat and rye, and Ni > Zn in wheat and triticale. The highest potential for PHE accumulation in cereals, on the basis of the calculated BC F1 values (Figure 2b), was observed for Ni > Sb > Cu in barley, Ni in maize, Tl > Cu > Sb in oat, Cu > Sb > Tl > Ni in rye, Ni > Cu > Tl for triticale, and Cu > Ni > Sb in wheat. The values of the calculated transfer factors are the function of PHEs in soils, and thus, they may vary in different locations; however, crops grown in the vicinity of mined non-ferrous metal ores should be monitored for potential contamination [21,58]. 

### 3.4. Human Health Risk Assessment

The results revealed the exceeded contents of Pb and Cd in reference to the MAC values. As to the remaining investigated PHEs, their safe concentrations in cereals were not specified. Moreover, the concentrations of PHEs in cereals are rather site specific and depend on the PHE concentration in soil. Finally, cereals are the main food consumed daily worldwide. Thus, considering the abovementioned reasons, the HHRA calculated in this study constituted an important part of our investigation. 

#### 3.4.1. Daily Intake Rates

According to available cereal consumption data [45], it was observed that a statistical Pole consumed 34.5 g of cereals daily (see Table 2). Because other statistical data were not available, the recommended daily intake rates of cereals by adults and children were adopted from the Australian Dietary Guidelines [4], equal to 585 g and 390 g, respectively. 

**Figure 3:**
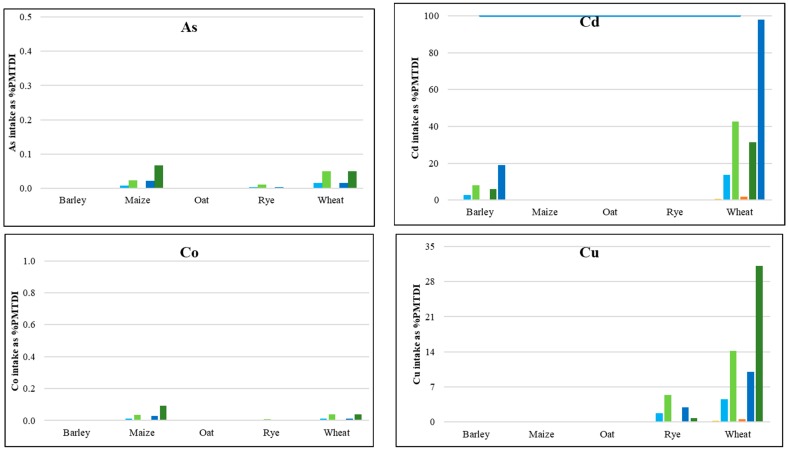

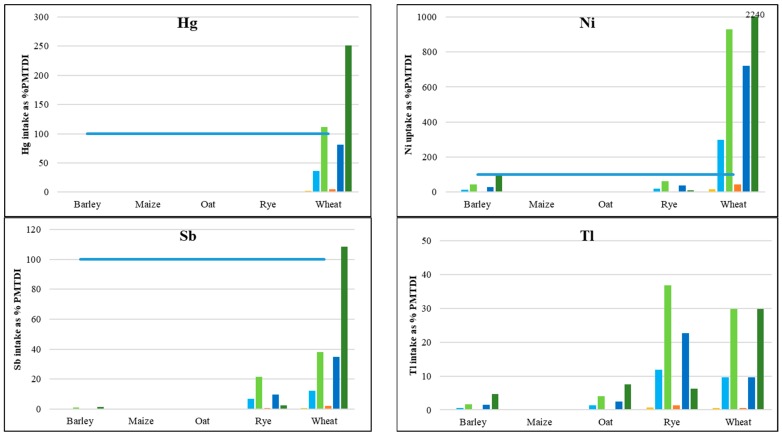

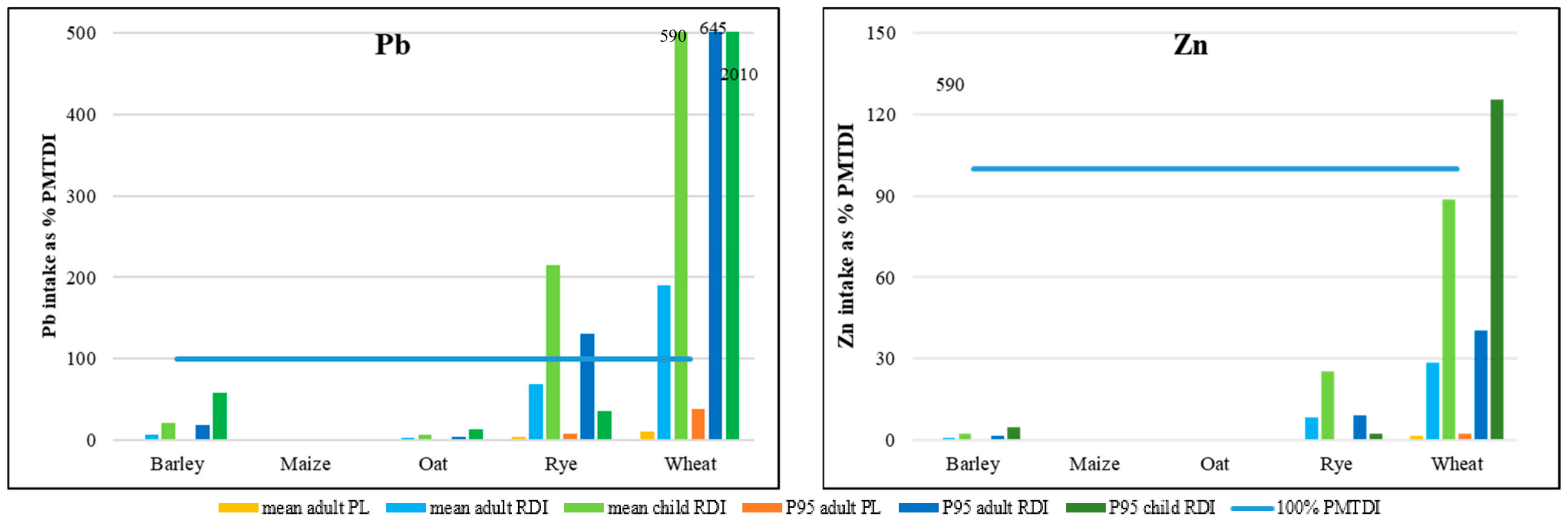
Daily intake rates of PHEs, with consumed cereals as a % of provisional maximum tolerable daily intake (%PMTDI); mean adult PL—daily intake rate for adults, statistical data for Poland [45], mean PHE concentration; mean adult RDI—recommended daily intake rate for adults [4], mean PHE concentration; mean child RDI—recommended daily intake rate for children [4], mean PHE concentration; P95 adult PL—intake rate for adults, statistical data for Poland [45], 95th percentile of PHE concentration; P95 adult RDI—recommended intake rate for adults [4], 95th percentile of PHE concentration; P95 child RDI—recommended intake rate for children [4], 95th percentile of PHE concentration.

The calculated daily intake rate values of the consumed PHEs in six species of cereals were compared to the tolerable daily intakes of trace elements recommended by the Joint FAO/WHO Expert Committee on Food Additives (JECFA), World Health Organization, and United States Environmental Protection Agency guidelines. Using the provisional maximum tolerable daily intakes (PMTDI), the following values were adopted in this study (mg/kg bw per day): As 0.0021 [59], Cd 0.0008 [60], Co 0.0014 [61], Cu 0.5 [62], Hg 0.0006 [63], Ni 0.005 [60], Sb 0.006 [64], Tl 0.00014 [65], and Zn 1 [62]. The PHE intake rates, calculated as a percentage of PMTDI (%PMTDI) (Figure 3), revealed that, as regards the statistical daily consumption at the mean PHE concentrations for all six cereals, the mean PHE intake was as follows (as % of PMTDI): As 0.0003, Cd 0.193, Co 0.0003, Cu 0.075, Hg 0.424, Ni 3.94, Pb 3.16, Sb 0.23, Tl 0.27, and Zn 0.44. Considering the recommended daily intakes of cereals, it was stated that PMTDIs were exceeded in wheat in children, in a decreasing order of PHEs as follows: Ni > Pb > Hg > Zn > Sb > Cd. The above-described observations are the effect of the fact that wheat is the main cereal cultivated in Poland and that the chemical composition of agricultural soils in southern Poland depends strongly on the geological background abundant in PHEs, as well as on intense anthropogenic activities in that area [29]. 

#### 3.4.2. Non-Carcinogenic Risk of PHEs

**Figure 4:**
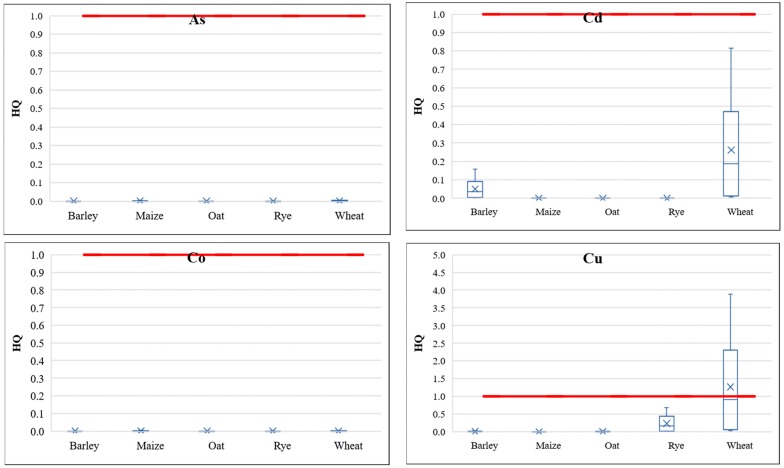

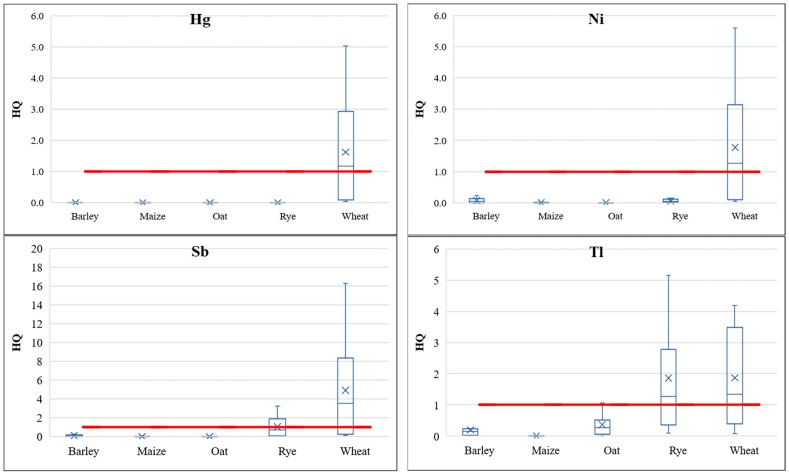

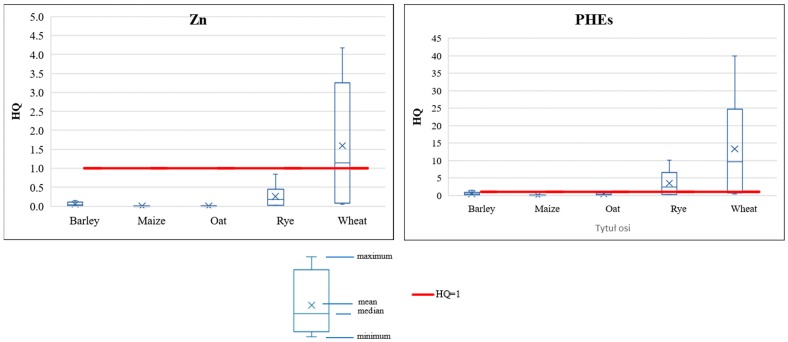
The non-carcinogenic risk values (HQ) of PHEs caused by cereal ingestion under the analyzed intake scenarios.

The target non-carcinogenic risk value was set to be equal to 1, according to the USEPA [43], as well as the Polish Regulation of the Minister of the Environment of 1 September 2016 on the conduct of the assessment of contamination of the surface of the Earth [66]. A statistical characterization of the calculated HQ for the respective cereals under the analyzed intake scenarios was presented in Figure 4. For particular PHEs, the target HQ=1 value was exceeded in wheat in the decreasing order of PHEs as follows: Sb (mean HQ = 4.91) > Tl (HQ = 1.87) > Ni (HQ = 1.77) > Hg (HQ = 1.62) > Zn (HQ = 1.59) > Cu (HQ = 1.27) and rye: Tl (HQ = 1.85) > Sb (HQ = 1.04). The total non-carcinogenic risk in the investigated cereals (as the sum of the HQ of single PHEs) revealed that risk was exceeded in wheat (mean HQ = 13.3) and rye (HQ = 3.44). For the remaining cereals, the total non-carcinogenic risk values were not exceeded and were ordered in the decreasing order of cereals as follows: barley (HQ = 0.47) > oat (HQ = 0.38) > maize (HQ = 0.02). As regards the statistical daily consumption of all cereals in Poland, the total non-carcinogenic risk value of the mean PHE contents in cereals was equal to 0.58, not exceeding the target risk value. At the 95th percentile of PHE contents in cereals, the total non-carcinogenic risk value was equal to 1.12, slightly exceeding the target risk value. 

#### 3.4.3. Carcinogenic Risk of PHEs

**Figure 5:**
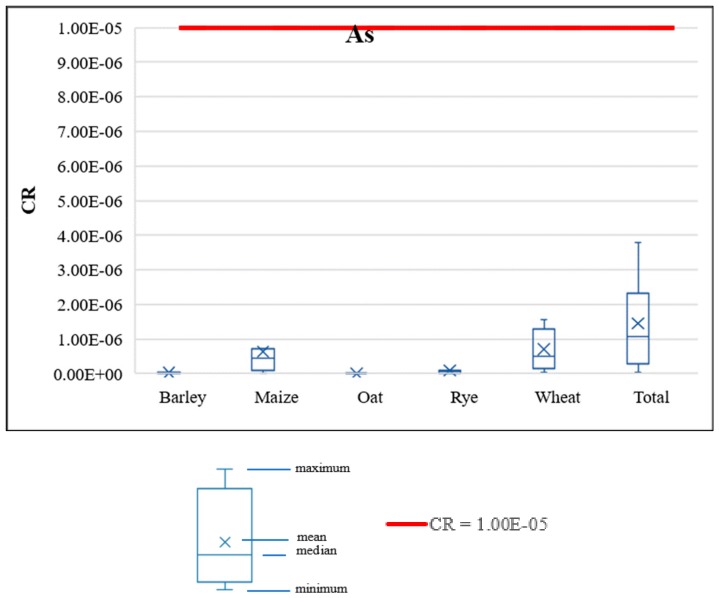
Carcinogenic risk (CR) values for As under the analyzed cereal intake scenarios.

The acceptable carcinogenic risk level was set to be equal to 1 × 10^−5^, as defined in the Polish Regulation of the Minister of the Environment of 1 September 2016 on the conduct of the assessment of contamination of the surface of the Earth [66]. In this study, only As was investigated as carcinogenic according to the current knowledge of the SF values for chemical substances. Although, As contents were determined only in maize samples, for other cereals, halves of the LOD values were assigned (see Section 2.4). The statistical characterization of the calculated CR values of the investigated cereals, under the analyzed intake scenarios, is presented in Figure 5. The acceptable CR level of As was not exceeded in any of the analyzed intake scenarios. As regards the statistical daily consumption of all cereals in Poland, the carcinogenic risk value of the mean As content in cereals was equal to 5.1 × 10^−8^, and at the 95th percentile of the As content in cereals, the carcinogenic risk value was equal to 7.8 × 10^−8^, not exceeding any acceptable levels in either case. 

#### 3.4.4. Margin of Exposure to Pb

The BMDL values are used to determine the health risk from exposure to Pb, although those values are not health-based guidance values but, rather, the levels below which health risk is considered to be acceptable low [17]. Thus, using the MOE approach, values <1 indicate high health risk, while values of MOE >1 point at an acceptable low risk. Although, Pb contents were not determined in the triticale samples in this case, LOD values were assigned (see Section 2.4).

Considering the statistical daily consumption of cereals in Poland, the MOE value for the mean Pb content was equal to 1.27, indicating an acceptable low risk; however, at the 95th percentile of Pb content, the MOE value was equal to 0.42, indicating a high risk of exposure to Pb (Table 5). Considering the recommended daily intakes of cereals by both adults and children, the MOE values were <1, indicating high risk arising from the Pb content in consumed cereals collected from the investigated regions of southern Poland. 

#### 3.4.5. Uncertainties in HHRA 

The value of calculated risk depended strongly on the respective intake rates. The risk values also depended strongly on the PHEs in the cereals cultivated in various regions of southern Poland, especially on the Pb contents in the Śląskie region, and that fact caused overestimation of the risk values for all of southern Poland. When the recommended amounts of cereals for daily consumption were used, they also caused overestimation of the calculated risk values, because specific recommendations were rarely followed. Again, it has to be stated that food recommendations have to be based on environmental standards. Moreover, the completed HHRA assumed that the investigated cereals constituted 100% of the PHE intake, excluding other ingestion sources, as well as other exposure pathways, i.e., dermal contact or inhalation. Taking the above into consideration, it should be stated that the HHRA presented here is only part of the whole risk assessment that may be calculated, although the goal of the present investigation was to assess if there was a potential threat posed by the PHE content in the consumed cereals in the area of southern Poland. 

## 4. Discussion

The contents of Pb, Ni, Zn, and Cd measured in this study in six investigated species of cereals, were lower, and the Cu contents were at the same level in wheat [1,12,18,19,22,23,26,27], maize [1,12,22,27], and barley [1,27]. That may have been associated with anthropogenic pollution of the irrigating water and soil via industrial production, mining, or transportation [12]. Those differences might also be associated with the cultivated area, climate, cultivation practice, or cultivars [67]. The results obtained by Olero et al. [25] indicated higher concentrations of As, Cd, Cr, and Pb in maize cultivated close to PHE emission sources, which were also reported by Shobha and Kalshetty [26] and Ibrahim et al. [68]. The concentrations of Pb, Zn, and Cd in barley were consistent with the results obtained by Eticha and Hymete [69], who reported that differences in metal contents in barley of different origins might be caused by differences in arable soil types, agrochemical treatment, and environmental pollution. The abovementioned results are in line with the observation that higher PHE concentrations in investigated cereals might be the consequence of the fact that Zn-Pb [70,71] and Cu [72,73] ores are present in the geological background of soils in southern Poland. Moreover, the contents of Pb and Cd reported in wheat and barley grains sampled in northern Poland [74], where non-ferrous geological deposits do not occur, were lower than those identified in this study. In reference, the PHE contents determined in this study were contained within the concentration ranges of cereals cultivated in Poland reported by Kabata-Pendias [5]. Because Cd, Ni, Zn, Pb, and Cu were present in bioavailable forms in the investigated soils of southern Poland [30], that might cause PHE accumulation in the analyzed cereals. The results obtained by Kan et al. [75] indicated the highest Zn contents in rye, which were in line with the results obtained in the present study. However, for other PHEs, significant differences were reported among cereal cultivars affected by various agricultural practices, soil types, types of fertilizers, and plant protection products [75]. Large differences in PHE contents in various cereal species were also reported between European and Asian countries, while PHE levels were rather similar among European countries [3]. 

Moreover, it has to be underlined that the results discussed here were based on investigations performed on 18 cereal samples that might not be sufficiently representative. However, the main goal of the presented research was to assess the human health risk for consumers based on reliable PHE contents in cereals cultivated in the analyzed region of southern Poland.

In addition, the PCA was performed on the results from only 18 samples. Generally, the higher the number of samples, the more precise the results [76]; however, an analysis performed on 19 samples revealed correct results in 90% of cases [77]. Thus, the PCA results obtained might have shown some inadequacy due to the investigated number of samples.

Apart from the general use of soil-to-plant transfer indices and knowing that PHE translocation from roots to seeds in the plant is generally very limited [78], in this research the transfer factor values were calculated to assess if the PHE amounts taken up by cereals did not cause their accumulation in consumed seeds. The calculated BA were <1, indicating that total PHE contents in investigated soils [29] (Table S1) were not taken up by cereal seeds in elevated amounts [24]. However, the calculated BC values were >1 for Ni, Zn, and Cu, as well as for Tl and Sb. This could be caused by the presence of these PHEs in such high amounts in potentially bioavailable forms in soils [30] (Table S1) that after translocation in plants, their contents in seeds were significant. Moreover, Ni and Zn content in cereal might be caused by the presence of these PHEs in the commonly used mineral fertilizers in Poland. Mineral fertilizers commonly used in Poland were found to be abundant in Ni and Zn, and elevated Ni contents were found in fertilizers with admixtures of grounded dolomite [79]. Organic manure used for fertilizing also contained significant amounts of Ni [80].

Considering the contents of PHEs in the investigated samples, limits of detection were used as the determined values and were lower than the limits of quantification (LOQ). However, as the main goal of the study was the health risk assessment, the PHE contents in the analyzed samples were necessary to obtain further results. Thus, as LOD values are the lowest concentrations likely to be reliably distinguished [81], this approach was intentionally used. It was recognized that the obtained results might have been unrepresentative; however, the performed research was done according to the parent rule of the conservative risk assessment principle.

The determination of the average daily intake is the critical point of the human health risk assessment analysis. In this research, permissible safe levels of PHEs in food were taken from the WHO as a global organization and from the USEPA as the author organization of the used methodology. However, there is not always compliance among health institutions with respect to the safe limits of PHE intakes in food. Moreover, the safe limits of food contaminants often change as new evidence for health safety is obtained. The European Food Safety Authority Panel on Contaminants in the Food Chain (CONTAM Panel) revealed that values established by the Joint FAO/WHO Expert Committee on Food Additives (JECFA) are no longer appropriate. For As, a range of benchmark doses at a lower confidence limit of 1% extra risk (BMDL_01_) with values between 0.3 and 8 μg/kg body weight per day was identified [82]. For inorganic Hg, the EFSA CONTAM Panel stayed in line with JECFA, establishing a tolerable weekly intake for inorganic mercury of 4 μg/kg bw [83]. For Ni, the EFSA CONTAM panel derived a tolerable daily intake of 2.8 μg Ni/kg bw from a lower 95% confidence limit for a benchmark dose at 10% extra risk (BMDL10) of 0.28 mg Ni /kg bw [84]. For Cd, the EFSA CONTAM panel nominated a tolerable weekly intake of 2.5 μg/kg bw to ensure sufficient protection of all consumers [85]. For Pb, the EFSA CONTAM panel concluded that the safe intake value was no longer appropriate, and as there was no evidence for a threshold for a number of critical endpoints, it was not appropriate to derive a permissible tolerable weekly intake (PTWI) [48]. In the case of essential PHEs, such as Cu [86] and Zn [87], the EFSA Panel on Dietetic Products, Nutrition, and Allergies (NDA) proposed daily adequate intakes depending on age and sex. Thus, comparisons of results of different HHRA investigations must be taken with caution, as they may lead to significantly different interpretations of risk values. 

## 5. Conclusions

The potentially harmful element contents in six cereal species cultivated on arable soils in southern Poland were investigated, and in reference to that, the human health risk implications were assessed as well. The contents of Pb and Cd significantly exceeded the MAC values in wheat, oat, rye, and barley in the Śląskie region. In maize, Pb and Cd contents were much lower than the MAC values. The bioaccumulation coefficient, calculated for the total PHE content in soil, indicated that cereals had no potential of PHE accumulation in seeds. The PHE daily intake rates of all six cereals based on the statistical daily consumption ranged from hundredths of a percent to a maximum of several percentage points of the permissible maximum tolerable daily intake. The calculated non-carcinogenic risk values for all the investigated PHEs were exceeded only in wheat (HQ = 13.3) and rye (HQ = 3.44). As regards the statistical daily consumption of cereals in Poland, the total non-carcinogenic risk was at the acceptable level according to the mean PHE contents. The acceptable cancer risk level of As was not exceeded under any of the intake scenarios; however, only the HHRA for As contents in maize was analyzed. Risk arising from Pb content in consumed cereals defined based on the MOE approach was acceptable low. The presented research proved that, even though the permissible levels of PHEs in soils have not been exceeded, monitoring of particular food crops is pivotal because the environment is getting more and more polluted, and all factors affecting the soil-to-plant transfer remain not fully known in the context of further consumption by animals and humans. Thus, it would be recommended to extend the current research on a larger scale with a statistically significant number of samples and computational modeling of the HHRA analysis to verify the reliability of the obtained results.

## Figures and Tables

**Table 1:** Description of the cereals investigated in this study.

No.	Common Name	Scientific Name	Number of Samples	Edible Part
1	Barley	*Hordeum vulgare*	3	seed
2	Maize	*Zea mays*	3	seed
3	Oat	*Avena sativa*	2	seed
4	Rye	*Secale cereale*	2	seed
5	Triticale	*×Triticosecale*	2	seed
6	Wheat	*Triticum aestivum*	6	seed

**Table 2:** Intake rates (IR), in g of wet weight (ww)/person per day, of the consumed species of cereals.

IR (g ww/Person Per Day)			
Type of Cereals	Adult PL	Adult RDI	Child RDI
Barley	0.78	13.3	8.85
Maize	0.16	2.69	1.79
Oat	0.09	1.58	1.05
Rye	5.68	96.4	64.4
Triticale	– *	– *	– *
Wheat	27.8	471	314
Sum	34.5	585	390

adult PL—intake rate for adults, statistical data for Poland [45]; adult RDI—recommended daily intake rate for adults [4]; child RDI—recommended daily intake rate for children [4]; *—no intake rate reported [45].

**Table 3:** Statistical parameters of potentially harmful elements (PHEs) in cereals.

PHE	Statistical Parameters	Barley (*n* = 3)	Maize (*n* = 3)	Oat (*n* = 2)	Rye (*n* = 2)	Triticale (*n* = 2)	Wheat (*n* = 6)	Cereals (*n* = 18)
As	min	ND	<LOD	ND	ND	ND	ND	<LOD
	mean ± SD		0.004 ± 0.002					0.001 ± 0.000
	max		0.013					0.013
	95th percentile		0.012					0.003
Cd	min	0.003	<LOD	ND	ND	<LOD	<LOD	<LOD
	mean ± SD	0.115 ± 0.003	0.006 ± 0.003			0.004 ± 0.002	0.017 ± 0.004	0.024 ± 0.002
	max	0.291	0.011			0.008	0.043	0.291
	95th percentile	0.267	0.011			0.008	0.039	0.091
Co	min	ND	<LOD	ND	ND	ND	ND	<LOD
	mean ± SD		0.004 ± 0.002					0.001 ± 0.000
	max		0.012					0.012
	95th percentile		0.011					0.003
Cu	min	1.09	0.002	1.20	1.66	1.28	0.11	0.002
	mean ± SD	1.42 ± 0.01	0.047 ± 0.01	4.56 ± 0.01	6.32 ± 0.14	1.42 ± 0.06	3.38 ± 0.10	2.93 ± 0.08
	max	1.57	0.97	7.97	11.0	1.57	8.10	11.0
	95th percentile	1.55	0.91	7.64	10.5	1.55	7.44	5.88
Hg	min	ND	<LOD	ND	ND	ND	<LOD	<LOD
	mean ± SD		0.002 ± 0.001				0.032 ± 0.011	0.006 ± 0.002
	max		0.006				0.080	0.080
	95th percentile		0.006				0.072	0.025
Ni	min	0.42	<LOD	0.11	<LOD	<LOD	<LOD	<LOD
	mean ± SD	3.68 ± 0.19	0.819 ± 0.086	0.49 ± 0.22	0.74 ± 0.08	1.29 ± 0.02	2.22 ± 0.04	1.54 ± 0.09
	max	8.40	1.484	0.86	1.47	2.58	5.86	8.40
	95th percentile	7.78	1.421	0.83	1.40	2.45	5.35	3.32
Pb	min	<LOD	<LOD	<LOD	<LOD	ND	<LOD	<LOD
	mean ± SD	2.17 ± 0.02	0.002 ± 0.001	6.00 ± 0.03	3.01 ± 0.08		1.69 ± 0.01	2.15 ± 0.02
	max	6.52	0.066	12.0	6.01		6.76	12.0
	95th percentile	5.87	0.060	11.4	5.71		5.75	5.25
Sb	min	0.047	<LOD	0.016	0.170	<LOD	<LOD	<LOD
	mean ± SD	0.099 ± 0.006	0.036 ± 0.012	0.071 ± 0.012	0.300 ± 0.022	0.011 ± 0.006	0.109 ± 0.015	0.104 ± 0.012
	max	0.152	0.108	0.126	0.430	0.021	0.351	0.430
	95th percentile	0.147	0.097	0.120	0.417	0.020	0.311	0.252
Tl	min	<LOD	<LOD	<LOD	<LOD	<LOD	<LOD	<LOD
	mean ± SD	0.004 ± 0.002	0.001 ± 0.000	0.080 ± 0.001	0.012 ± 0.003	0.007 ± 0.001	0.002 ± 0.001	0.018 ± 0.001
	max	0.012	0.001	0.160	0.024	0.013	0.002	0.160
	95th percentile	0.011	0.001	0.152	0.023	0.011	0.002	0.063
Zn	min	33.5	5.47	43.2	51.2	38.8	21.2	5.47
	mean ± SD	43.3 ± 1.54	7.40 ± 1.16	44.9 ± 1.11	59.4 ± 2.01	40.2 ± 2.12	42.3 ± 0.54	39.6 ± 1.41
	max	50.1	8.39	46.7	67.7	41.5	60.4	67.7
	95th percentile	79.7	8.38	46.5	66.8	41.4	59.9	55.8

LOD the limit of detection; ND < LOD in all analyzed samples.

**Table 4:** Factor loading results obtained from the principal component analysis (PCA) for the cereals samples.

PHEs	Varimax Rotated		
	PC1	PC2	PC3
As	**0.897**	0.305	0.079
Cd	−0.103	**−0.815**	−0.372
Co	**0.897**	0.305	0.079
Cu	**−0.827**	0.443	0.317
Hg	−0.122	−0.314	0.498
Ni	−0.281	**−0.937**	−0.146
Pb	**−0.730**	0.419	−0.403
Sb	**−0.611**	0.138	0.565
Tl	−0.455	**0.609**	**−0.604**
Zn	**−0.964**	−0.104	0.125
Eigenvalues	4.44	2.60	1.38
Explained variance %	44.4	26.0	13.8
Cumulative variance %	44.4	70.4	84.2

Factor loadings >0.6 or <−0.6 are shown in **bold.**

**Table 5:** Margin of exposure (MOE) values for Pb under the analyzed cereal intake scenarios.

Cereals Intake Scenario	MOE (Mean Exposure) ^1^	MOE (P95 Exposure) ^2^
adult PL	1.27	0.42
adult RDI	0.07	0.03
child RDI	0.01	0.005

P95—95th percentile; adult PL—intake rate for adults, statistical data for Poland [45]; adult RDI—recommended daily intake rate for adults [4]; child RDI—recommended daily intake rate for children [4]; ^1^—calculated for mean Pb content in investigated cereals; ^2^—calculated for P95 Pb content in investigated cereals.

## Supplementary Materials

The following are available online at https://www.mdpi.com/1660-4601/17/5/1674/s1, Table S1: PHE contents in arable soils in southern Poland (based on [1,2]) used for soil-to-plant transfer indices in this study, Figure S1. Ordination diagram of PCA, computed for the PHE contents in cereals samples. Figure S2. Dendogram of PHEs in cereals, according to Sneath’s criteria. Figure S3. The color-scale map of standardized PHE contents in cereals species.
