# Supplementary material for: Human Health Risk Assessment and Potentially Harmful Element Contents in the Cereals Cultivated on Agricultural Soils
Source: Int J Environ Res Public Health. 2020 Mar 4;17(5):1674. doi: 10.3390/ijerph17051674 (PMC7084233; doi:10.3390/ijerph17051674)
Supplement: Supplementary file 1 [file ijerph-17-01674-s001.pdf]

International Journal of
Environmental Research and Public Health

MDPI

Supplementary material

Human health risk assessment and potentially harmful element contents in the cereals cultivated on agricultural soils

Agnieszka Gruszecka-Kosowska [1*]

[1] AGH University of Science and Technology, Faculty of Geology, Geophysics, and Environmental Protection, Department of Environmental Protection, Al. Mickiewicza 30, 30-059 Kraków, Poland; agnieszka.gruszecka@agh.edu.pl
ORCID 0000-0002-4988-173X

* Correspondence: agnieszka.gruszecka@agh.edu.pl (A.G-K.)

Table S1. PHE contents in arable soils in southern Poland (based on [1,2]) used for soil-to-plant transfer indices in this study (modified from [3]).

Figure S1. Ordination diagram of PCA, computed for the PHE contents in cereals samples.

Figure S2. Dendogram of PHEs in cereals, according to Sneath's criteria.

Figure S3. The color-scale map of standardized PHE contents in cereals species.

Table S1. PHE contents in arable soils in southern Poland (based on [1,2]) used for soil-to-plant transfer indices in this study (modified from [3]).

PHEs	Mean total PHE contents in arable soils analyzed in southern Poland [1]	Mean PHE contents in exchangeable and acid soluble forms in arable soils analyzed in southern Poland [2]	Mean PHE contents in potentially soluble forms present in pore water in arable soils analyzed in southern Poland [2]
	extraction with aqua regia	extraction with 0.11 mol/dm^3 CH_3COOH in first step of the BCR sequential extraction procedure	extraction with 0.05 mol/dm^3 Na_2EDTA
		mg/kg dw.	
As	6.64	0.28	na
Cd	0.39	0.45	0.72
Co	4.92	0.44	na
Cu	26.6	0.72	4.45
Ni	11.5	0.64	1.29
Pb	63.8	<LOD	21.3
Sb	1.23	0.04	na
Tl	0.1	0.005	na
Zn	283	63.6	40.3

PHEs—potentially harmful elements, BCR—Community Bureau of Reference, dw.—dry weight.

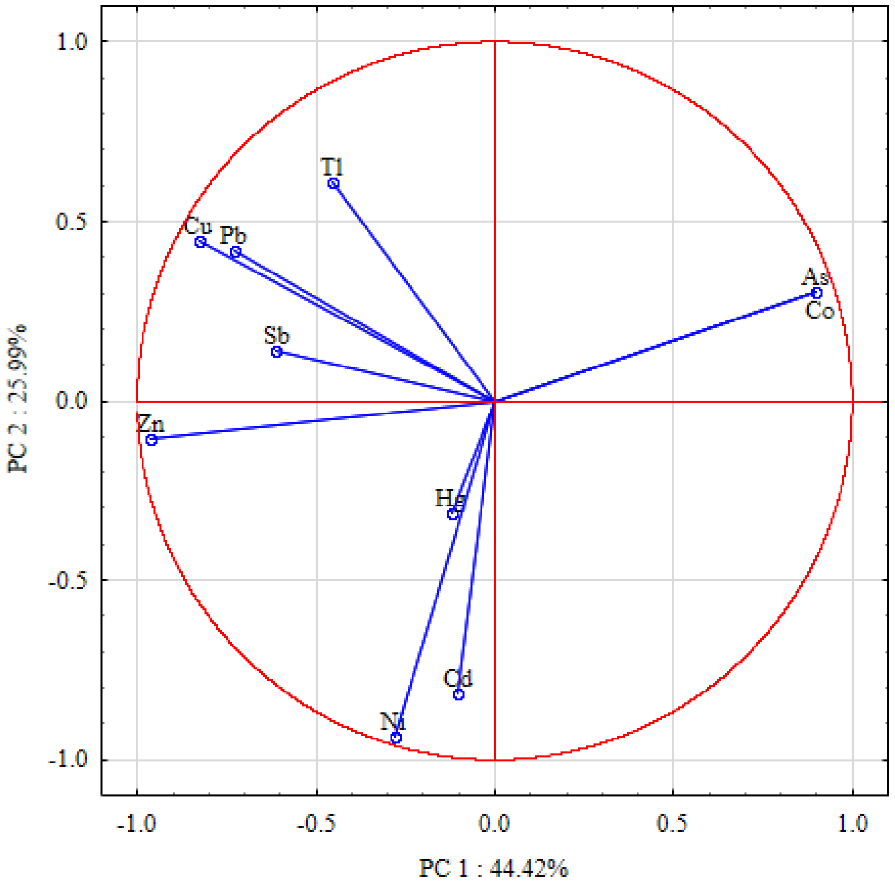

Figure S1. Ordination diagram of PCA, computed for the PHE contents in cereals samples.

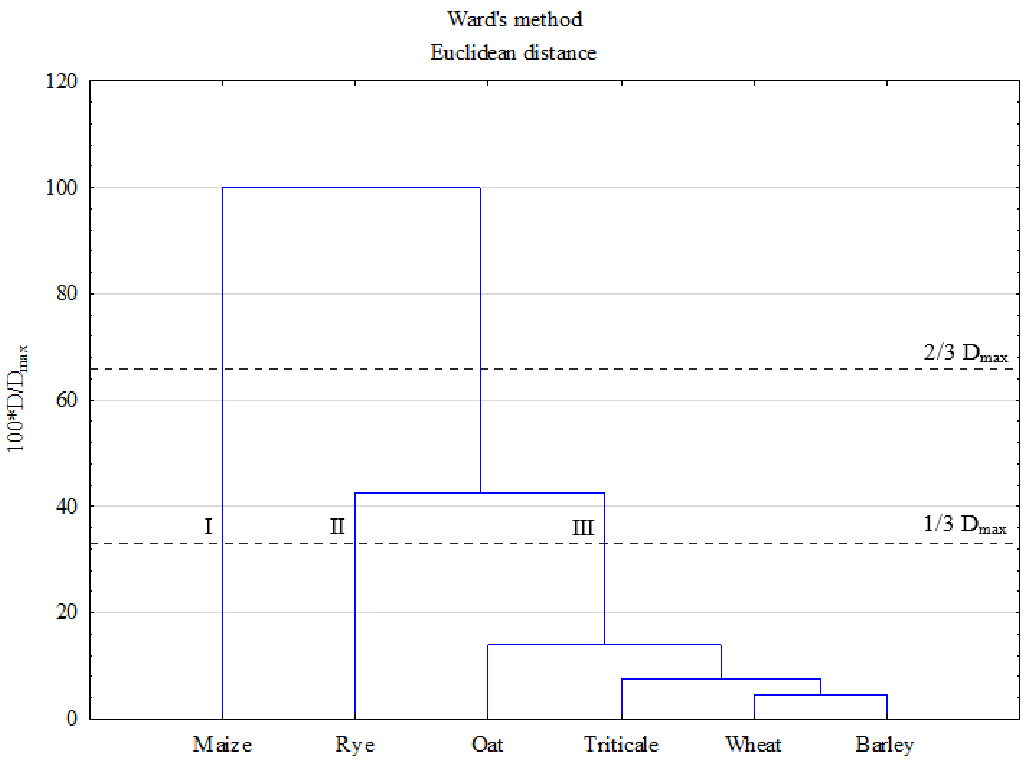

Figure S2. Dendogram of PHEs in cereals, according to Sneath's criteria.

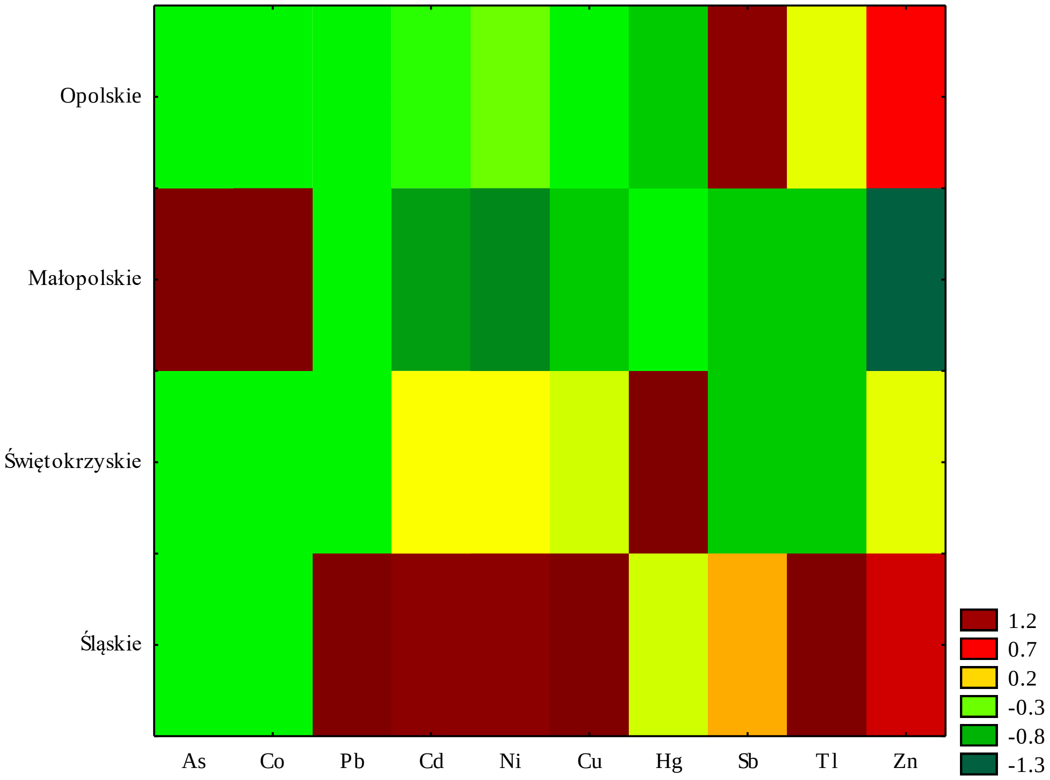

Figure S3. The color-scale map of standardized PHE contents in cereals species.

References

1. Gruszecka-Kosowska, A.; Baran, A.; Wdowin, M.; Mazur-Kajta, K.; Czech, T. The contents of the Potentially Harmful Elements in the arable soils of southern Poland, with the assessment of ecological and health risks: A case study. *Environ. Geochem. Health* **2019**, doi.org/10.1007/s10653-019-00372-w.
2. Gruszecka-Kosowska, A.; Baran, A.; Mazur-Kajta, K.; Czech, T. Geochemical fractions of the agricultural soils of southern Poland and the assessment of the Potentially Harmful Element mobility. *Minerals* **2019**, *9*, 674.
3. Gruszecka-Kosowska, A. Human health risk assessment and potentially harmful element contents in the fruits cultivated in the southern Poland. *Int. J. Environ. Res. Public Health* **2019**, *16*, 5096.

© 2019 by the authors. Submitted for possible open access publication under the terms and conditions of the Creative Commons Attribution (CC BY) license (http://creativecommons.org/licenses/by/4.0/).
